# A significant exploration on meta-heuristic based approaches for optimization in the waste management route problems

**DOI:** 10.1038/s41598-024-64133-1

**Published:** 2024-06-27

**Authors:** Gauri Thakur, Ashok Pal, Nitin Mittal, Mohd Shukri Ab Yajid, Fikreselam Gared

**Affiliations:** 1 Department of Mathematics, Chandigarh University, Ajitgarh, India; 2 Department of Industry 4.0, Shri Vishwakarma Skill University, Palwal, Haryana India; 3 Management and Science University, Shah Alam, Selangor Malaysia; 4 Faculty of Electrical and Computer Engineering, Bahir Dar University, Bahir Dar, Ethiopia

**Keywords:** Metaheuristic, Routing, Optimization, Waste management, Bibliometric, Environmental sciences, Engineering

## Abstract

In metropolitan cities, it is very complicated to govern the optimum routes for garbage collection vehicles due to high waste production and very dense population. Furthermore, wrongly designed routes are the source of wasting time, fuel and other resources in the collection of municipal trash procedure. The Vehicle Routing Problem (VRP) published between 2011 and 2023 was systematically analysed. The majority of the surveyed research compute the waste collecting problems using metaheuristic approaches. This manuscript serves two purposes: first, categorising the VRP and its variants in the field of waste collection; second, examining the role played by most of the metaheuristics in the solution of the VRP problems for a waste collection. Three case study of Asia continent has been analysed and the results show that the metaheuristic algorithms have the capability in providing good results for large-scale data. Lastly, some promising paths ranging from highlighting research gap to future scope are drawn to encourage researchers to conduct their research work in the field of waste management route problems.

## Introduction

According to the form of substance or nomenclature, solid waste is the undesirable by product of manufacturing methods, community or home chores, and is sometimes denoted as junk, rubbish, trash or garbage. Due to its harmful results on the environment, solid waste management has grown to be a significant environmental problem. The procedures of generation, collection, transport, handling, value retrieval, and succeeding disposal comprise solid waste management. Any of these processes with poor design incur higher operational costs and risk environmental contamination^1^. For instance, the assortment and transportation procedure alone are responsible for 60–80% for the overall cost of solid waste management (SWM)^2^. Management organisations will be considerably impacted by ineffective SWC (solid waste collection) and transport because it will increase operational costs and ultimately lower profit. If sustainable SWM in developing economies is to be accomplished, garbage collection and transportation costs must be reduced. As a result, Oduro-Kwarteng^3^ proposes system study and operational optimization to ensure well-organized and effective SWC. Therefore, SWC and transportation should be carried out in a method that will provide both cost savings and environmental preservation.

In the early years, complex combinatorial optimization problems like the VRP were often solved using exact methods or specialised heuristics. Then, more general solution techniques were developed and named metaheuristics by Fred Glover in 1986^4^. The difficulty comes from adapting those general solution approaches to the current problems. Metaheuristics, as opposed to traditional heuristics, conduct a more complete exploration of the solution space, permitting weak and frequently unsustainable actions in addition to recombining solutions to produce novel ones. Consequently, although requiring more computing time than early heuristics, meta-heuristics can consistently produce solutions of a high quality. Due to their efficiency in solving challenging optimization problems, metaheuristics are a preferred approach in numerous applications. Metaheuristics are a category of optimization strategies that guide the search process to get better results. When it is not possible to build an explicit equation-based model, they are especially helpful. Population-based algorithms are employed in metaheuristic optimization to address a wide range of optimization problems in numerous fields such as internet routing, robot path planning, networking, image processing and engineering design. Real-world problem optimization is frequently characterized by its complexity, non-linearity, multiple conflicting goals, and challenging constraints. It can be difficult to find the best answer for such problems can be an arduous task, as optimal solutions may not even exist in some cases.

In the domain of optimization, a task that comprises minimization or maximization can be formulated as a problem:

1 $$Find X=\{{x}_{1},{x}_{2,}...{x}_{n}\} which\,minimize/maximize f(X)$$

subject to

$${g}_{j}(X)\le 0,\,\,j=\text{1,2},...,m$$

$${l}_{j}\left(X\right)=0,\,\,j=\text{1,2},...,p,$$

where $f(X)$ is the objective function, design vector is the n-dimensional vector X, ${g}_{j}(X)$ signifies inequality constraints and ${l}_{j}(X)$ signifies equality constraints respectively. The goal of this study is to analyse metaheuristic optimization for the solution for VRP problems in SWM. A metaheuristic implementation can also deliver nearly ideal results in a manageable amount of time. Particularly for VRPs, there is a remarkable track record of metaheuristic implementation success.

SWM involves many variables including the waste bin’s location, collection points, vehicle capacities, travel distances, and time windows. The objective of VRP is to optimize the routes and schedules for waste collection vehicles to minimize costs while satisfying constraints. Metaheuristics provides an efficient solution to tackle the complexity of such optimization problems. By applying metaheuristic algorithms to solve the VRP, waste collection routes can be optimized to minimize fuel consumption, reduce vehicle wear and tear, optimize crew schedules, and enhance the overall operational efficiency. Metaheuristic techniques can help decision-makers assign restricted resources (such as vehicles, manpower, and time) effectively in waste management operations. By optimizing routing and scheduling decisions, these techniques allow better resource utilization and cost savings. Waste management operations frequently face dynamic and undefined situations such as variations in waste generation rates, traffic congestion, and road closures. Metaheuristic algorithms offer robustness and adaptability to handle such uncertainties by continuously adjusting routes and schedules based on real-time information. Optimizing waste collection routes using metaheuristic algorithms can contribute to environmental sustainability by reducing greenhouse gas emissions, minimizing vehicle miles travelled, and encouraging effective resource utilization.

The above motivates to analyse, metaheuristic techniques for VRP in SWM for providing insights into how these advanced optimization techniques can address the challenges faced by waste management systems, leading to more efficient and sustainable waste collection operations. The amount of research addressing the VRP and waste management route optimization combined is insufficient, despite the fact that both topics are broadly studied in the literature. Metaheuristics are widely used since VRP can’t be accurately computed in the long run. Based on the performance and specified difficulties experienced during the gathering of trash, this analysis examines the VRP through metaheuristic algorithms for solving SWM. WMP by VRP is an important and evolving study field, so there is a lot of space for advancement. But before anything further can be done, a review of the problem description and the approach taken by earlier researchers in this field is necessary. In this study, metaheuristics approaches are analysed by bibliometric survey including three case studies have been showed for encountering future scope.

By taking these introductory remarks into consideration this paper is structured as follows. “Literature survey” includes the literature survey, survey methodology is explained in “Survey methodology”, work is discussed in “Discussion on the work analysed”, “Case studies in Asia” includes the analyses of three case studies. “Discussion on case studies” includes the discussion on case studies and “Findings and challenges” is concluded with future scope.

## Literature survey

This section involves three parts. First, Mathematical programming approaches towards waste collection problem. Second, VRP and its variants literature for waste collection problem (WCP) and in last metaheuristic with routing approach are briefed. Besides, waste collection studies that are handled by VRP and metaheuristic algorithms too surveyed.

**Figure 1:**
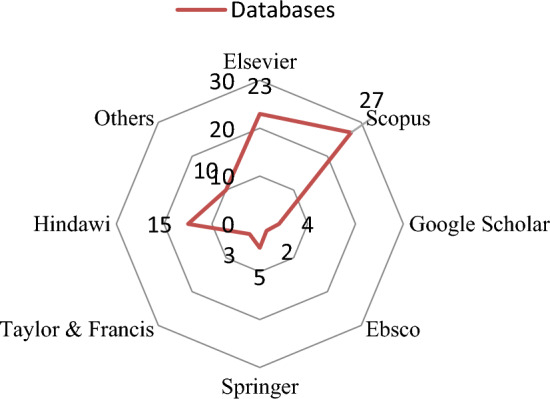
Databases where literature have been acquired.

Peer-reviewed books, papers, and journal articles totalling 387 were studied for analysing literature survey. Figure 1. shows the details of primary databases for retrieving the articles. literature review's keywords included SWC systems, waste management, the VRP, optimum system designs, and metaheuristic algorithms. After reviewing each article, 104 appropriate papers were chosen for the literature review.

### Mathematical programming towards waste collection problem

The WMS often entails garbage collection, waste transit to any intermediate location or to a disposal site, as well as waste recycling and waste treatment^5^. Our study mainly focuses on waste transportation problem. It lacks scientific planning and research and is a weak association in the solid waste environmental management scheme. This has resulted in significant loss of labour, material resources, financial resources, and usable resources. Based on the fundamental conditions in our nation, the optimal route for garbage vehicle is being developed^6^. Numerous studies using mathematical modelling have been conducted as a result of the demand for the most efficient routing of vehicle for the assemblage and delivery of waste. The first article in 1959 on the "truck dispatching" problem by Dantzig and Ramser and a lot has changed since then^7^.

Clark and Gillean^8^ in 1975 proposed the simulation system to address the complex issues with the solid WMS, which resulted in a decrease in the yearly budget from $14.8 million in 1970 to $8.8 million in 1972 and the number of employees from 1640 to 850. The enhancement of a SWC system was presented by Ronen et al.^9^ in 1983. They used a heuristic model to analyse and modify the garbage collection routes, and when the planned routes were implemented, one out of every six compilation teams were spared, and the overall distance covered was cut by 18.7%. In 2000, Sonesson^10^ proposed the garbage collection model to determine fuel usage and time. Make use of basic statistics to simulate and reasonably forecast real outcomes, with an energy consumption deviation of between 5 and 14% and a time consumption deviation of between 10 and 24%. In order to recommend a suitable routing system, Awad and Aboul-Ela^11^ solved the Chinese Postman Problem (CPP) and the Travelling Salesman Problem (TSP) by means of Monte Carlo simulation, heuristic algorithm in 2001. Ghiani et al.^12^ in 2005, proposed the study on SWC for the municipality’s health sector of Castro-villari, Italy. The author utilizes real arc routing problem for achieving the decrease in 8% of entire cost. In 2013, Naninja^13^ discussed how to formulate an integer programming problem (ILP) and solve it using LP software to optimise the transport charge of solid waste by minimising the number of vehicle journey. In 2015, Das and Bhattacharyya^14^ proposed an ideal gathering and transportation arrangement which concentrates for the issue of reducing the span of individual collection and transport route by mixed integer programme as a heuristic approach. Mehdi et al., and Amir et al.^15^ in 2021, A probabilistic stochastic mathematical programming model is created. The ε-constraint technique has been used to solve and validate the model. Additionally, a case study using the proposed approach is put into practise in Qazvin, Iran, for real-world applicability.

Researchers have used a number of mathematical schemes to optimise the collecting and transportation routes for vehicles that collects garbage. According to Ronen et al.^9^, these models can only manage a portion of the constraints that the collection routing difficulty entails and only provide partial solutions when used practically.

### VRS and its variants approach towards waste management

One of the awful difficult operational complications in developing nations is waste collection^16^. In the collection of solid waste, wastes are gathered from several divided areas and transported to intermediary facilities^16^. Once entire gathering points have been visited or the routing requirements have been satisfied, a collection vehicle departs from the depot, begins assembling garbage from the customers, when the vehicle is occupied, it arrives at the midway facility for the unloading procedure. At the end, complete vehicles should go back to the depot empty after unloading waste at intermediate facilities^17^. But in some circumstances provided the unloading constraint and methods are followed, the unloading operation can be completed the following day. In these situations, a vehicle leaves the depot to transport waste to an assemblage point and then moves on to the following assemblage point. At the end day, it goes back to the depot with the waste, and the next day, the vehicle is emptied at the disposal site.

The VRP is one of the general ways to control garbage collection. J. Caceres-Cruz et al.^16^ states that the objective of VRP is to improve routes while abiding by all relevant limitations, including capacity, time window, vehicle count, and garbage. The routing difficulties is significant because it deals by time and cost restrictions, scheduling, and meeting consumer needs. Dantzig and Ramser first discussed vehicle routing in 1959, and it has since become a significant topic of research^7^. 15% of the cost of goods sold and one third to two thirds of the company's logistical expenditures are often attributed to transportation. Logistics costs can be reduced by effectively handling the vehicle routing problem^18^. When it comes to waste collection, VRP lessens the number of trips completed, the distance covered, the amount of fuel used, and the emissions produced by the vehicles^19,20^. One of the earliest issues with waste collection and the routing difficulty were initially presented by Beltrami and Bodin^21^ (1974) for the Washington and New York City municipalities, together through a range of automobiles. They enhanced the ground-breaking method developed by Clarke and Wright^22^ in 1964 to identify the best paths. Their work's outcome was put to use in the municipalities and had an immense impact.

Capacitated VRP (CVRP), one of the fundamental VRP variant, determines the set of routes for a fleet of similar vehicles with the overall lowest route cost while satisfying all objectives. The VRP with Time Windows (VRPTW), which mandates that each vehicle deliver the goods to the clients within a particular time window (see Table 1). There are two types of time windows: VRP with Soft Time Window (VRPSTW) and VRP with Hard Time Window (VRPHTW). The vehicles' various capacities are assumed by the heterogeneous VRP (HVRP). The Multi-depot VRP (MDVRP) is predicated on the notion that a company may have a number of depots from which it can serve its clients. The Periodic VRP (PVRP), in which clients may be visited more than once, generalizes the traditional VRP by extending the planning horizon to several days. The Open VRP (OVRP), in which vehicles finish their delivery runs without necessarily going back to the original depot. If so, they must go to the same clients in the reverse sequence. The Time-dependent VRP (TDVRP), which is used to plan vehicle routing, assumes that travel speeds (times) depend on the time of travel. Hazardous Waste Collection VRP with Multiple Time Windows (HWCVRPmTW) is the variant for ensuring safety with all the efficient factors which comprises in waste collection. In the Stochastic VRP (SVRP) numerous components of the problem are random that follows a probability distribution. SVRP can be divided into the VRP with Stochastic Demand (VRPSD), the VRP with Stochastic Customers (VRPSC).

**Table 1:** Analysing VRP and its variants in WMS.

References	Author	Year	Technique	Highlights	Findings
^23^	D. Otoo et al	2016	VRPTW	Application of the suggested method to a real issue in Tafo Pankrono. Correlated to the local waste management company's regular collection schedule	Drastic 39% reduction in collecting time from the current collection time
^24^	Katja Buhrkal et al	2012	VRPTW	Regulating the appropriate efficient routes for garbage assemblage trucks, so that every trash can be unloaded Trash is transported to dumping site at the same time maintaining client time windows and making sure that drivers receive the necessary rest intervals	The approach produces better outcomes for benchmark cases. A novel real-world case was tested, and significant advancements were made
^25^	Intaek Gong et al	2020	VRPSDP-TW	Optimizing delivery and pickup schedules Extending the general VRP of time-windowed simultaneous delivery and pickup during accounting for leftover costs	Reduces the total delivery cost. Proposed method is applied to the several logistics businesses that need reusable transport dumpsters like shipping containers, refrigerating containers
^26^	B.-I. Kim et al	2006	VRPTW	Accounted for the drivers' lunch break and several disposal trips Algorithm for extended insertion a VRPTW algorithm for clustering-based garbage collection is created	The top provider of complete trash management services in North America, with almost 26,000 assemblage and transfer vehicles, successfully applied for the waste collecting problems
^27^	Aya Ishigaki	2016	SVRP	By taking demand volatility into account, model the dynamic collection strategy and created a search algorithm	Calculated the assigning of n consumers to K automobiles as a combinatorial optimization problem. The suggested search scheme is implemented to the garbage assemblage problem
^28^	Mariagraziaotoli et al.	2017	HWCVRPmTW	Maximize the amount of rubbish assembled on each trip while minimising the total distance travelled overall in an effort to meet the requests made, and the resulting profit for the business	Helps planners of the HWC by restricting the travel distance by road and by conducting what-if analysis under various scenarios to assess the benefits of any fleet investment. A true case study demonstrates the approach's efficacy
^29^	Han et al.	2015	VRPB	Waste assemblage classification (commercial, residential and industrial) WCVRP was formerly represented by node routing and arc routing problems	Minimizing travel distance, logistics expenses, and time to increase the effectiveness of collecting practices. Serves as a guide of research written in the area of WCVRP
^30^	Maurizio Faccio et al.	2011	WCVRP	It is feasible to choose which bins should be emptied and which should not based on the waste creation pattern by getting into the realistic information of individual vehicle and retrieval degree at individual bin. maximising a variety of factors	Introduces a cutting-edge vehicle routing model that is coupled with traceability data, describing an outline for the traceability technology accessible for finding optimal solution of SWC. Its implication is launched in an Italian city with a population of roughly 100,000. Simulations are used to verify and validate the model, and the paper's conclusion reports the results of an economic feasibility assessment
^31^	Hashimoto et al.	2006	VRPTW	Employed local search to regulate the vehicles paths deciding the route of each vehicle, computed the optimum beginning time of services for visited customers	Introduced travelling cost functions to broaden the restrictions on journey time for the VRP. On improved Solomon's benchmark instances, computational studies show the value of relaxing the time window and traveling time restrictions

### Metaheuristic algorithms for VRP in waste management problem

In recent years, several metaheuristics for the VRP have been published. These are generic problem-solving techniques that search the problem space for viable answers and frequently incorporate well-known heuristics for route design and improvement. In contrast to conventional techniques, metaheuristics allow for deteriorating and occasionally even unfeasible intermediate solutions during the search process. Despite being more time-consuming, the most eminent metaheuristics created for the VRP often recognize improved local optima than previous heuristics. Several metaheuristic algorithms are made for computing diverse forms of optimization problem. Some of them are, GA, TS, BA, ACO, SA, EA, CS, FA, PSO, HS etc.^32^.

The resulting Table 2. shows that how the algorithms are extremely diverse and have gained attention recently as workable solutions for a variety of VRP situations. According to the outcome analysis, the following trends metaheuristics are used: The data shows that the most frequently used methods for solving out these VRP variants are EC and GA techniques. DE is one of the oldest algorithms, although it is utilised relatively sparingly. HS, BA, and CS are not that much employed. The most popular SI algorithms are ACO and PSO. We have taken only eight VRP variants but there are many which are not included such as Fleet size and mix VRP (FSMVRP), VRP with route balancing (VRPRB), Reliable selective VRP (RSVRP), VRP with backhauls (VRPB), Dynamic VRP (DVRP), Risk constrained Cash-in-Transit VRP (RCTVRP), multi-depot green VRP (MDGVRP), VRP with heterogeneous fleet, mixed backhauls, and time windows (HVRPMBTW) and many more. GA is one of the algorithm which is used in enormous amount as compared to other metaheuristic algorithms but the scope never ends there are many variants of VRP which is left out by GA and they can become base of future research in this area. ABC, GWO, IWD, CS SFLA, GSO, CoEA, DA and FA are used in very less amount. Therefore, we find that future study could concentrate on testing the performance of the rarely used algorithms on other problem types. Future research has a lot of interesting opportunities with the underutilised algorithms. Finally, the generated classification tables are particularly useful for identifying potential areas for future metaheuristic and VRP problem research. The effort to include performance of employed metaheuristics as a categorization characteristic is ongoing.

**Table 2:** Several metaheuristic algorithms for solving different VRP variants.

Reference	Algorithm	CVRP	VRPTW	HVRP	MDVRP	OVRP	TDVRP	PVRP	VRPSD
Nazif and Lee^33^, Márquez et al.^34^, Du et al.^35^, Yu et al.^36^, Vidal et al.^37^	GA	✔	✔		✔	✔		✔	
Mirhassani et al.^38^, Xu et al.^39^, Du et al.^35^, Xu et al.^40^, Marinakis et al.^41^	PSO	✔	✔		✔	✔			✔
Fleming et al.^42^, Yu et al.^36^, Du et al.^35^	ACO	✔	✔		✔				
Cao et al.^43^, Pavel Krömer et al.^44^	DE					✔			✔
Marinakis and Marinaki^45,46^	BBMO					✔			✔
Fleming et al.^42^, Yassen et al.^47^, Du et al.^39^, Kuo^48^, Goodson et al.^49^	SA	✔	✔		✔		✔		✔
Qi et al.^50^, Chen et al.^51^	MA		✔						✔
Szeto et al.^52^, Zhang et al.^53^	ABC	✔	✔						
Marinaki and Marinakis^54^	GSO								✔
Chen and Liu^55^, Luo and Chen^56^	SFLA		✔		✔				
Teymourian et al.^57^	CS	✔							
R. Yesodha; T. Amudha^58^, Alinaghian and Naderipour^59^	FA	✔					✔		
Teymourian et al.^57^	IWD	✔							
L Korayem et al.^60^, Diastivena et al.^61^	GWO	✔			✔				
De Oliveira et al.^62^	CoEA				✔				

### Research gaps

Research gaps in the literature on TSP, VRP, and its variants VRPTW, VRPSD, HVRP, OVRP, and CVRP in waste management route optimization frequently concentrate on general routing problems without taking waste-specific constraints into account, such as the frequency of waste collection and the need for specialized vehicles to handle various waste types (e.g., hazardous materials, recyclables). In order to increase the efficiency of waste collection operations, there is a research gap in the development of routing models and algorithms that explicitly take these waste-specific restrictions into consideration. Many existing methods rely on static routing scenarios and fail to sufficiently account for dynamic factors including fluctuating rates of waste generation, congestion, road closures, and weather conditions. Research on dynamic and real-time optimization strategies is required in order to improve operational efficiency and responsiveness by adapting waste collection routes in response to changing circumstances. The application of advanced technologies, such as the Internet of Things (IoT), vehicle telematics, GPS, and Geographic Information System (GIS), offers opportunities to improve waste management's route optimization. However, current studies frequently underutilize these technologies or fall short of realizing their full potential in order to improve route design and implementation. The study of novel strategies that make use of innovative technologies for vehicle tracking, real-time data collecting, route optimization, and performance monitoring in waste management operations is lacking.

Many research works on metaheuristic-based VRP waste management solutions focus on particular parts of the world, such North America or Europe. Studies on other regions, especially those in Asia, Africa, Australia, and Latin America, might be lacking. Investigating the performance of metaheuristic algorithms under various environmental and infrastructure conditions, as well as in various geographic contexts, may yield insightful information.

In addition to reducing the number of bins that must be visited and emptied, the combination of exact data with the multi-object vehicle routing structure also has a direct positive influence on environmental factors like discharges, noise, and road traffic crowding. The majority of studies focus solely on reducing expenses or travel time. Although they are uncommon, the environment and service quality are seen as important considerations. Additional goals like increasing service quality or lowering emissions could be added, making the study in the WCP field multi-objective. Studies with multiple objectives can make a substantial contribution to solve both the economic and environmental problems. Heuristic and metaheuristic methods are more appropriate for larger datasets than exact methods are for smaller datasets. The capacity of approximation approaches to get accurate results for massive amounts of data is one of its strongest points. As a result, there is a growing pattern of trends in waste collection VRP employing benchmark data and case studies. Addressing these research gaps can contribute to advancing the state-of-the-art in metaheuristic based VRP solutions for waste management, leading to more effective, robust, sustainable, and resilient waste collection route system.

## Survey methodology

Recent years have seen a significant increase in interest among researchers in bibliometric analysis. It not only offers a stand-alone platform for an area's general progress, but it also makes room for potential future research. Additionally, rather than getting lost in the multitude of articles, young researchers might choose a beginning point for their own research. There have been a number of these investigations in significant study fields. In recent times, Muhuri et al.^63^ investigated Industry 4.0's bibliometric component and analysed related prior research. The thorough bibliometric analysis of type-2 fuzzy sets and systems was provided by Shukla et al.^64^. Yu and Shi^65^ investigated the intuitionistic fuzzy set of Atanassov in this way with the use of citation analysis. Real-time operating schemes by Shukla et al.^66^, Green supply chains by Amirbagheri et al.^67^, Energy proficiency by Trianni et al.^68^, and others are some more key research areas with bibliometric studies. Applied soft computing by Muhuri et al.^69^, Engineering applications of artificial intelligence by Shukla et al.^70^, IEEE transactions on fuzzy systems by Yu et al.^71^, Knowledge-based systems by Cobo et al.^72^, European journal of operational research by Laengle et al.^73^, these are just a few journals that include bibliometric analysis as well.

At the outset, a wide set of academic studies, Web of Science database was employed to accumulate data on the use of metaheuristic algorithm for VRP in waste collection. The databases were examined by means of metaheuristic algorithm, vehicle routing, waste management, waste collection in the search expression. The possibilities for the "Subject/Title/Abstract" box were searched for this exact phrase. Due to this restriction, less irrelevant results were found as well as results where the metaheuristic algorithm for VRP was mentioned but not specifically examined. The methodology employed in this study is thoroughly explained in this part, along with how papers were retrieved, categorised, and distilled.

### Statistical observations

**Figure 2:**
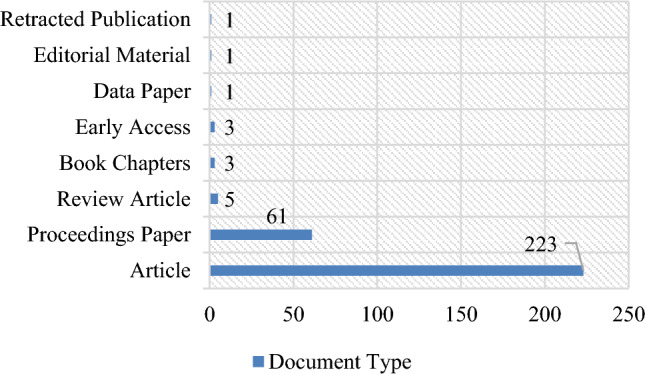
Listing of document type in retrieved literature.

**Figure 3:**
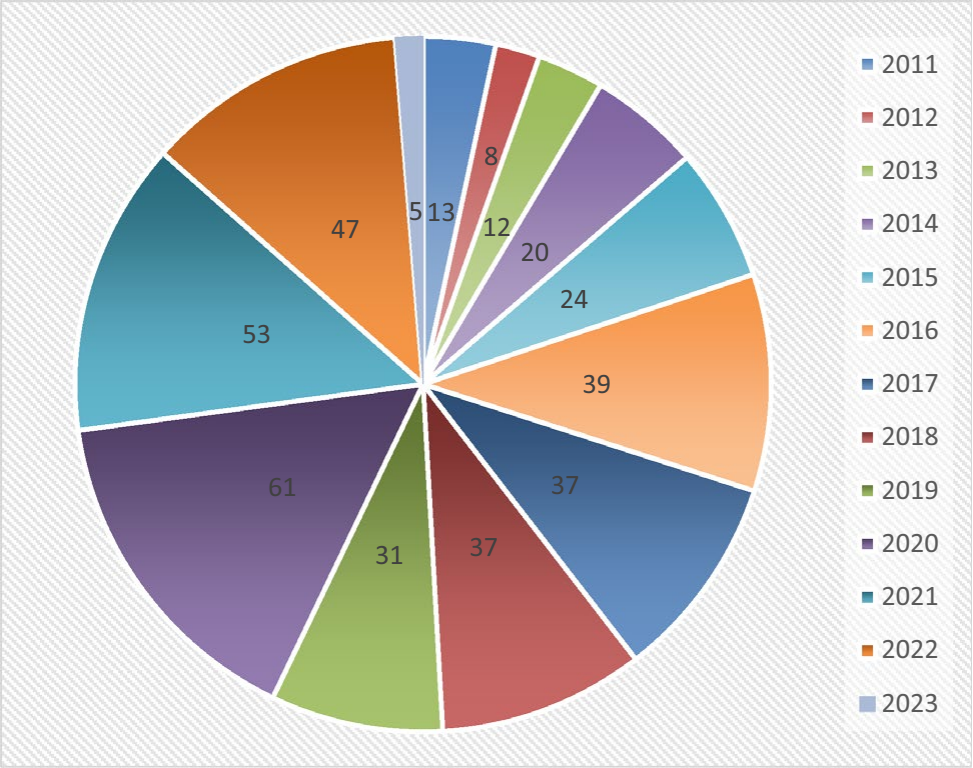
Annual Scientific Production (2011–2023).

The 387 bibliographical entities, which included journal articles, book chapters, and articles from several conference sessions, were retained for preliminary analysis. A breakdown of the compiled bibliography from 2011 to 2023 is shown in Fig. 2. Even though we have complete access to the majority of the 223 journal articles listed above, some of them are either inaccessible or only have an abstract available. Geographical barriers to accessing regional journals and access restrictions on publisher databases like Springer and EBSCO are the main causes of this inaccessibility. 198 of the journal articles are fully accessible to us, while 58 are only accessible with limited access. The following 5 articles are only accessible as bibliographical data. In past decade, role of metaheuristic algorithms has been increased in the field of VRP. Figure 3. Shows the annual scientific production of metaheuristic algorithms in VRP for waste collection problems. The literature progress rate is nearly perfectly exponential with a 12. 39% annual scientific growth rate. This fact alone proves metaheuristic algorithms and VRP’s vitality in waste collection problem. Though, it is not as speedy when associated to other coexistent decision-making development disciplines. So, this field requires much more research.

### Structural analysis

**Figure 4:**
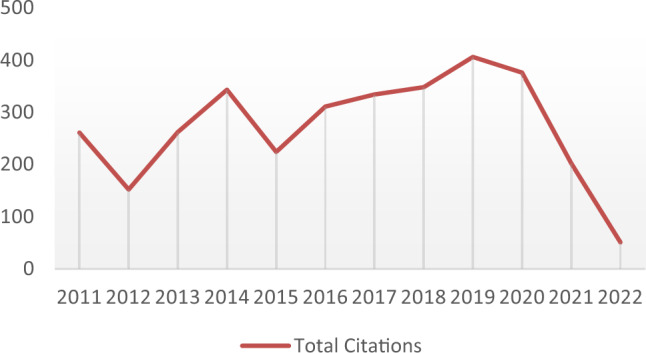
Analysis of metaheuristic algorithm for VRP in waste management.

In Table 3, the journals are arranged in descendant order of the most cited 25 sources for the number articles published from 2011 to 2023. From this it can be observed that Waste management, Journal of cleaner production, Waste management and research, Computers and operation researcher by far the journals of preference for the authors. Waste management, Journal of cleaner production, Waste management and Research Science together account for 20% of all metaheuristic and VRP for waste collection articles published in refereed journals. In the year 2012 and 2015 there was a downfall with 11% and from 2016 the figure peaked at with 30% growth and from the year 2018 to 2020 the growth was increased exponentially with 1130 total number of articles. In 2019 the there was a decrease of 9%. The way a study field uses citations is another trend worth examining. The line graph of citations received by articles published in the WMS domain is shown in Fig. 4. Between 2011 and 2023, there were 1117 authors in total. There were only 3238 citations of the work in this field, as can be seen from the number of publications.

**Table 3:** Listing the analysed articles with respect to most cited academic journals.

Title	Articles
Waste Management	19
Journal of Cleaner Production	13
Waste Management & Research	12
Computers & Operations Research	9
European Journal of Operational Research	9
International Journal of Environmental Research and Public Health	7
Journal of the Operational Research Society	7
Sustainability	6
Transportation Research Part E-logistics and Transportation Review	6
Annals of Operations Research	5
Computers & Industrial Engineering	5
Expert Systems with Applications	5
Transportation Science	5
Applied Sciences-Basel	4
Applied Soft Computing	4
International Transactions in Operational Research	4
Mathematical Problems in Engineering	4
Networks	4
Operational Research	4
Resources Conservation and Recycling	4
Sensors	4
Environmental Monitoring and Assessment	3
IEEE Internet of Things Journal	3
IEEE Transactions on Intelligent Transportation Systems	3
Journal of Heuristics	3

### Analysis of metaheuristic algorithms for WCP

There are many different VRP output techniques which are commonly examined in the work done till now. Dynamic programming, which makes use of mathematical models, and algorithms intended to expand the models are examples of exact approaches. Simple heuristics and metaheuristics are two categories of heuristic algorithms. Metaheuristics are called problem independent approaches which attempts to retain good superiority solutions by lower handing out times in problematic optimization situations, in contrast to simple metaheuristics that depend on the problem at hand. Simple heuristics typically attempt to create a workable solution before trying to enhance it. Some of the most popular simple heuristics include the algorithms Sweep^74^, Christofides^75^, Nearest Neighbour, and Improved Petal^76^. Contrarily, metaheuristics efficiently search the solution space by enhancing basic heuristics and combining other techniques^77^. A synopsis of each research contribution, particularly in terms of their suggested strategies for the real world as well as the recorded advancement, is given in Table 4. According to additional constraints, algorithm, objective function, and vehicle type, Tables 4 and 5 provide an overview of the garbage collection solutions using metaheuristic optimization approaches.

**Table 4:** Implementation of metaheuristic algorithm to WCP.

References	Author	Algorithm	Location	Outcome
^78^	Gomez et al., 2015	Tabu Search	North-western Spain	MOAMP approach outperformed the current solution by minimizing transportation costs while maximizing service levels
^79^	Son, 2014	Chaotic PSO	Danang, Vietnam	Increased the total quantities of collected waste, but with increased travel distance and operational time
^80^	Huang and Lin, 2015	Ant colony optimization (ACO)	Kaohsiung, Taiwan	While the overall distance travelled remained constant, the frequency of close encounters increased on average by 68.97%
^81^	Assaf and Saleh, 2017	Genetic algorithm (GA)	Southern Nablus City in West Bank, Palestine	Total travel distance was lowered by 66%, total assemblage time was decreased from 7 h per truck travel to 2.3 h per truck travel
^82^	Xue and Cao, 2016	ACO	Singapore	Numerical value is not specified. Selecting the best route to deliver waste to the intended incinerator plant is an operational concern that waste collectors must regularly address. The model is a helpful tool in providing answers to these questions. Additionally, rather than depending just on drivers' potentially biased views, the model offers a quantitative-subjective method of answering this query
^83^	Nevrly et al., 2019	GA	Jihlava, Czech Republic	Total travel distance was reduced by 7%
^84^	Tirkolaee et al., 2019	SA	Sanandaj, Iran	Total cost was reduced by 13.3%
^85^	G. Rossit et al., 2021	SA	Argentinean city, Bahía Blanca	Shows better performance in both real MSW instances and benchmark instances than the other metaheuristics including CPLEX
^86^	Elshaboury et al., 2021	ANN-PSO	Poland	The ANN-PSO scheme demonstrates its effectiveness at approximating the quantities of municipal solid trash and may be viewed as a practical way to create integrated waste management systems
^87^	Shan Jiang, 2020	WOA-PSO	Anshan, China	Good evaluation capabilities, and a good guiding significance for choosing the location of a waste incineration plant
^88^	Yu et al., 2022	GA-SA	Bandung City, Indonesia	The model is designed to account for the minimal number of depot facilities that each region is required by law to have. Establish the depot locations in each region and the routes taken by the trucks to collect waste in order to meet independent needs across regions at the lowest possible overall cost

**Table 5:** Briefing of metaheuristic optimization methods built on problem characteristics.

Category	References
Metaheuristic algorithm	
Ant colony optimization	^80,82,89,90^
Constructive Heuristic	^91–93^
Genetic algorithm	^81,83,88,94–98,104^
Particle swarm optimization	^79,87,98,99^
Simulated annealing	^84,88,101^
Tabu search	^78,102^
Whale optimization algorithm	^87,103,104^
Objective function	
Minimize distance	^83,87,89,91,95,105^
Multi-objective	^78–82,84,92,94–101,103,106^
Additional constraint	
Multiple depot	^88,96,97^
Multiple dump sites	^106,107^
Time-dependent	^59,91,96^
Time windows	^34,39,50,84,105,106^

The following articles present the analysis of the methodologies employed in Table 4.

Gomez et al.^78^ proposed Tabu search technique for a bi-objective urban waste collection problem. The two objectives were lowering the cost of transportation and maximizing the standard of service. More specifically, the frequency of waste collection at each point during the planning period determines the level of service. Within the framework of multi-objective adaptive memory programming (MOAMP), a solution strategy for this problem was established by employing tabu search, and the outcomes were compared with an implementation of NSGA-II, a well-known multi-objective optimization technique. Tabu search (TS) is an iterative neighbourhood search algorithm, where the neighbourhood changes dynamically. Tabu search enhances local search by avoiding points in the search space which are already visited. By avoiding already visited points, loops in search space are avoided and local optima can be escaped. This problem required a schedule of daily routes over a lengthy planning period. Two decision levels were taken into account: (1) the small villages that needed to be visited each day, and (2) the layout of the daily routes that went with them. The main contribution is the adaptation of the MOAMP framework to this problem, which finds nondominated solutions and approximates the efficient frontier by employing tabu search as the engine and performed experiments with a set of artificial and real instances. The performance of MOAMP and the widely used NSGA-II approach were compared in the experiments and also compared the findings with the authorities' present approaches. The experiments highlighted the advantages of the proposed strategy.

Son^79^ presents an efficient optimization technique for the municipal SWC problem at Danang city. An innovative vehicle routing model was created with the goal of maximizing the total amount of waste that all vehicles collected. The hybrid approach was proposed based on the model that combines elements of chaotic particle swarm optimization and the binary gravitational search algorithm to identify a set of feasible options using ArcGIS software to select the best option displayed on a map interface. The proposed strategy has been implemented into in ArcGIS and could provide the best possible planning outcomes, such as the total amount of waste that vehicles had gathered, equivalent routes, vehicle travel distances, and total operational time. The proposed technique outperforms other related methods, including the manual collecting procedure already in use in this city, according to experimental validation conducted on the real dataset of Danang.

Huang and Lin^80^ present the development of an ACO algorithm that features route improvement to minimize vehicle use and distance travelled, as well as to guarantee that the time interval between the two consecutive services for any block is not too short and minimum cost model for the municipal WCP on Taiwan's "Keep Trash off the Ground" policy. In order to verify the improved performance of this modified algorithm in the multi-trip SDVRP with pickup and delivery, the ACO was compared and with the route improvement in this study. Simulation and optimization findings show that the ACO algorithm with route the improvement outperforms the ACO alone by a significant margin. The modelling of a real-time vehicle routing system and the effective application of the ACO to address this problem are the contributions of this study to the literature. The foundation for recognizing the potential for raising the quality of service provided by municipal SWC may be laid by this study, which could be extremely significant.

Assaf and Saleh^81^ proposed optimizing the VRP for the MSW collection problem in Nablus city located in the northern West Bank in Palestine. The existing SWC system in the southern Nablus region is based solely on truck drivers' experience, which has resulted in high operating expenses and repeated trips to some dumpsters while some are skipped. Creating a mathematical model that reduces the overall distance driven by the MSW collection vehicles is the main goal of this study. Because there are a lot of dumpsters in the city's southern area, the researchers used GA to find the model's optimal solution. In order to achieve this, a mathematical model for a capacitated vehicle routing problem (CVRP) was developed and applied to the SWC problem in the city's southern region. The shortest route for each vehicle (truck) was then found using GA, these shortest routes ensure that each dumpster is visited just once during the journey. The approach can be utilized in a reasonable amount of time to identify almost ideal solutions, even though the solution identified is static and cannot be employed in the event of certain unexpected events (such as a truck going out of service or the driver being absent). According to the authors, the municipality ought to establish a distinct section tasked with managing and optimizing the SWC problem using regularly collected data.

Xue and Cao^82^ proposed routing model, which is an adaptation of the ACO coupling with min–max model and Dijkstra’s algorithm which is capable of solving the multi-objective shortest path problem (MOSP) based on the Pareto front. The loosely linking model can bridge the gap created by these models independently and optimize the strengths of these various models. The suggested model is better in exploring the pareto front solution space due to the adaptive weights setting design. By preventing the ants from creating a path when they create a loop, the proposed approach also introduces a novel technique to increase the algorithm's efficiency in solving these kinds of MOSPs. The case study has illustrated the suggested model's ability to enable routing decision-making in the context of Singapore's waste collection system in addition to demonstrating the model's ability to find the best routes on the pareto front.

Nevrly et al.^83^ proposed the integration of GA and local search as the heuristic algorithm for waste collection in arc routing problem. To accurately represent the situation, tasks pertaining to collecting waste, fleet sizing, container location, and evaluation of the total economic and environmental impact must be completed in accordance with operational specifications. The main goal of using defined tasks is to minimize expenses and their negative effects on the environment, population, and investment decision-making. The Jihlava real network was used to test the proposed algorithm. There are 3529 edges and 1467 vertices in the considered network. Additional input parameters, such as vehicle capacity and operating costs, production, and demand for waste collection, were suitably generated based on estimations from waste management experts and demographic data. The maximum number of people for both feasible and infeasible scenarios was fixed at 100. Iteration average for calculation time was 0.69 s. A diversification process is introduced when the solution has not been improved for 30 iterations which adds diversity to the population and thus revives the entire crossover process. The real case study involved 8594 nodes and was carried out for a different city. The calculation was carried out for a particular district and route, where 160 bins for the collection of paper waste were connected to 264 arcs, including inverse arcs. Savings were found when the output was compared to the route that is currently in use. The length of the algorithm suggested path is 66.7 km, however the actual trip is 71.4 km. This indicates a possible savings of about 7%, or 1400 EUR annually for a single route.

Tirkolaee et al.^84^ proposed a mixed-integer linear programming (MILP) model for heterogeneous multi-trip vehicle routing problem with time windows specific to the urban waste collection, which goal is to find the best service routes and the optimum number of the used vehicles. Based on the problem's taken into consideration, a stimulated annealing (SA) algorithm is created to solve it. The SA is used to enhance the solutions, and this algorithm is used to independently enhance each of the initial solutions. SA is a local search algorithm that has the capability to escape from local optimums. When compared to the CPLEX solver, the results of solving the problem in small and medium sizes show that this algorithm provides nearly optimal solutions. Lastly, a case study is examined to determine whether the suggested model can be used in real-world situations. It has been shown through analysis of the case study that the suggested model can enhance the current situation by producing a 13.3% reduction in the overall cost.

Rossit et al.^85^ presented the comparative study between the exact resolution and three metaheuristic solution tools to solve the problem of MSW collection in Bahía Blanca. The exact solution is based on a linear programming formulation of the CVRP model using the CPLEX software. In contrast, a simulated annealing algorithm (SA) is presented and compared with a genetic algorithm (GA) and an algorithm based on large neighbourhood search (LNS) from the literature. Furthermore, a comparative analysis of several metaheuristics is conducted with a few instances of a popular benchmark from the literature. The proposed SA significantly outperforms the conventional GA and performs similarly to the LNS algorithm, often generating values that are near to the optimal solution. Upon examination of the conducted experiments, it is confirmed that the use of these algorithms yields potentially satisfactory and proficient outcomes, with the benefit of metaheuristics requiring less computational resources to solve the problem. Based on the experience with benchmark problems and Bahía Blanca municipal solid waste instances, the suggested SA algorithm performed well when compared to alternative metaheuristics and CPLEX in both benchmark and real municipal solid waste instances.

Elshaboury et al.^86^ presented an ANN model coupled with the PSO algorithm and conventional ANN to forecast MSW quantities in Polish cities. The performance of the models was compared using five assessment metrics, namely, coefficient of efficiency (CE), Pearson correlation coefficient (R), Willmott’s index of agreement (WI), root mean squared error (RMSE), and mean bias error (MBE). To represent the effect of economic, demographic, and social factors on the rate of waste generation, selected explanatory factors were integrated into the developed models. Population, employment-to-population ratio, revenue per capita, number of entities by type of commercial activity, and number of businesses enlisted in REGON per 10,000 people were the considered criteria. The ANN–PSO model (CE = 0.92, R = 0.96, WI = 0.98, RMSE = 11,342.74, and MBE = 6548.55) surpassed the standard ANN model (CE = 0.11, R = 0.68, WI = 0.78, RMSE = 38,571.68, and MBE = 30,652.04), according to the findings.

Shan Jiang^87^ proposed the technique for addressing MSW incineration plants by adopting a merging of the particle swarm optimization (PSO) algorithm and whale optimization algorithm (WOA). These algorithms are swarm optimization algorithms, which search for the best fitness value through mutual collaboration between individuals in a swarm. The waste incinerator plant siting model based on the intelligent swarm optimization algorithm has a good evaluation capability and can combine a wide range of factors, including cost, policy, and environmental concerns, as demonstrated by the real results. The model has significant regional applicability and may be expanded to include other criteria based on the real circumstances, making it a useful tool for selecting the location of waste incineration plants.

Yu et al.^88^ proposed a novel variant of the location routing problem (LRP), named regional location routing problem (RLRP) and multi-depot regional location routing problem (MRLRP), by considering the minimum number of depots for each region and two types of depots and proposes two mathematical models and a hybrid algorithm of GA and SA named GASA. In addition, a new set of instances is created using a real-life study taken from PD Kebersihan in Bandung City in order to give a more precise illustration of the waste problem. The proposed algorithm's effectiveness in resolving RLRP and MRLPR instances is examined. GASA achieves close to optimal outcomes for the RLRP dataset, with the largest gap being 0.11% when compared to BKS (best solution offered GA and GASA). With a difference of 0.07% from BKS, GASA has the largest gap in the MRLRP dataset. In comparison to the commercial solution, the findings show that the proposed approach yields comparable results and a reasonable computing time.

By summarizing the methodologies and analysis employed as mentioned above it is being concluded that the multi-objective optimization with high-dimension are compatible for addressing waste routing problems because they allow decision makers optimize multiple conflicting objectives at the same time. The multi-objective optimization objective is to recognize a set of Pareto-optimal solutions, where no solution can be improved in one objective without lowering performance in another, which provides decision makers with a variety of trade-off possibilities, permitting them to make decisions based on their preferences and significances. The waste management depend on precise information on waste generation charges, collection points, road networks, and other aspects. An incomplete or inaccurate information can hamper the progress and execution of optimization approaches for waste collection. The implementing optimization strategies in waste collection may necessitate noteworthy investments in technology, such as GPS tracking systems, routing software, and data analytics techniques. The waste collection operations can have social and environmental effects beyond the goals of cost minimization and service quality maximization. These may comprise concerns associated to traffic congestion, noise pollution, air quality, and equity in service provision. The balance of these considerations with optimization objectives can be complex and may need participant role in decision-making processes.

**Figure 5:**
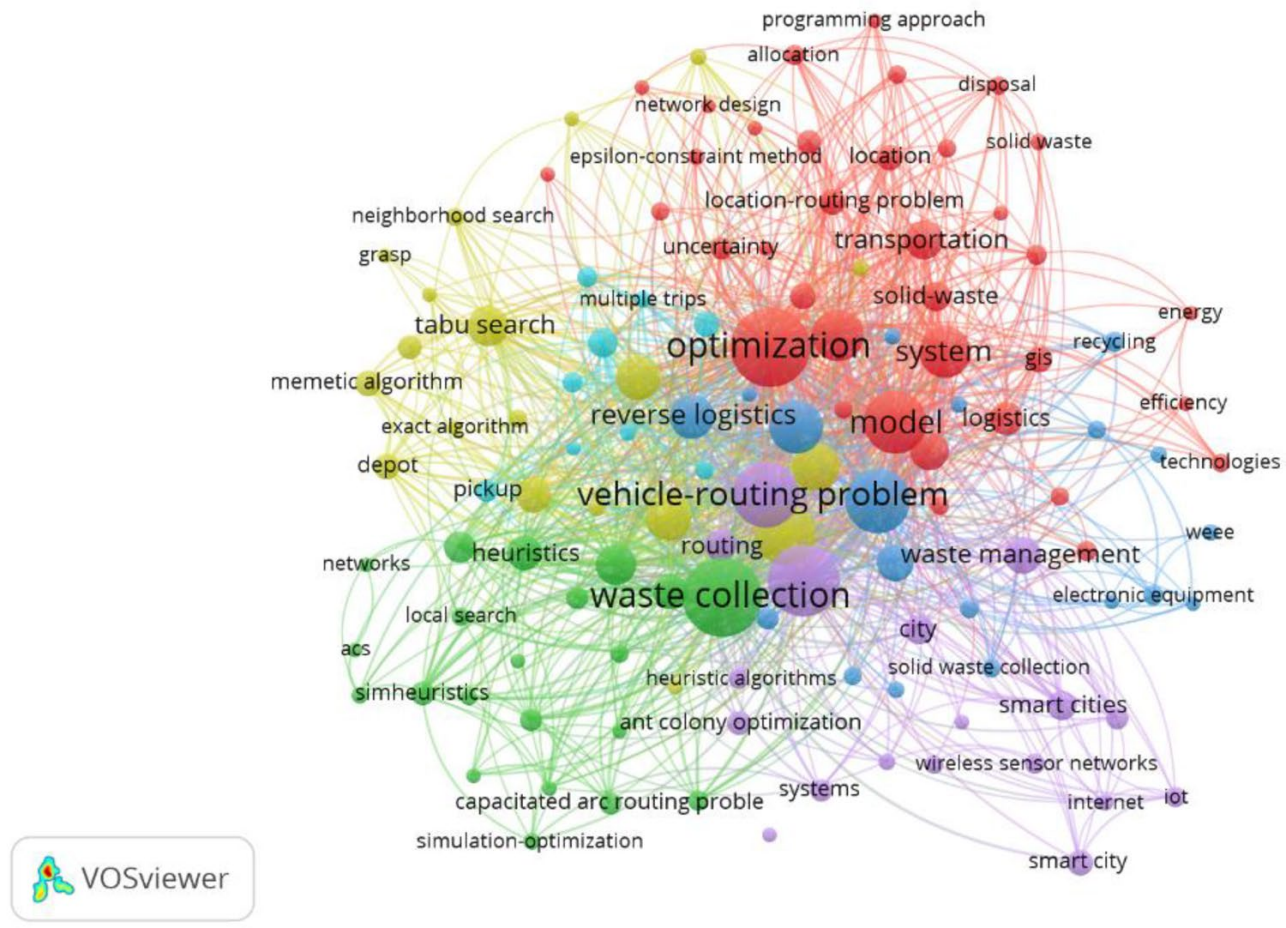
Visualization of metaheuristic algorithms for WMS.

**Figure 6:**
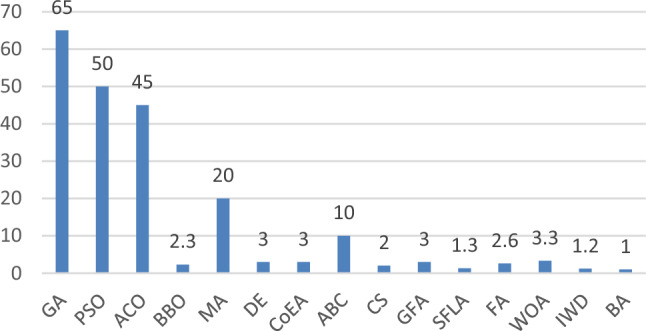
Overall percentage of occurrence of EC and SI algorithms in reviewed articles.

Figure 5. present visualizations of the cooccurrence of the metaheuristic algorithm in relation to VRP for waste management problem. Circles and labels are used to depict problem-specific domains. Each algorithm's weight or publication count is shown by the circle and size of the label on the graph for each related problem type. The connections between the candidate method, the VRP and metaheuristic version, and the type of problem being addressed are represented by the network lines. The graph's colours are used to show which groups of algorithms a given problem belongs to. Despite being a more modern algorithm than the GA, PSO, and DE, the network graphs of the WOA, GWO, FA, and CS algorithm nevertheless show a minor relevant impact of the application to WCP^108^. Figure 6 shows overall percentage of EA and SI algorithm in the reviewed articles. Despite this finding, GA, ACO and PSO were chosen as the representative algorithm due to their ability to explore and utilise some of their latent performance potential but the scope never ends there is much more need of metaheuristic algorithms in solving WCP. IOT or intelligent transport system can become barrier in the field of metaheuristic algorithm and VRP.

## Discussion on the work analysed

Research contributions on this subject have grown since the end of the 1990s as more and more scholars have realised the significance of waste management through VRP. Through the use of a metaheuristic algorithm and VRP literature, this work has made an effort to present a roadmap for WMS, along with some of its limitations and consequences. The collection of solid waste (organic and recyclable) is the main topic of this study. Other particular wastes, such as lubricating oil wastes, light wastes, and battery wastes, that also need to be gathered in a selected manner were outside the purview of this work and, as a result, which are not examined in this analysis. In coming years, the research might include additional wastes. This research study's analysis of the literature is limited to scholarly papers acquired from databases some of them are web of science, Ebsco, Scopus. This paper does not incorporate any other sources such as the newspapers, periodicals or technical reports.

In general, the VRP for waste collection is typically more challenging to resolve than a standard VRP. The metaheuristics approach to study has emerged as the maximum capable direction for the VRP family of complications, according to the literature examined in this work. Metaheuristics, as opposed to classical heuristics, conduct extra complete exploration of the solution space, permitting poor or occasionally infeasible actions along with recombining solutions to produce novel ones. As an outcome, although requiring more computing time than early heuristics, meta-heuristics can consistently produce answers of a high calibre. The two of the WCVRP benchmark difficulties are frequently utilized to evaluate systems. One is a benchmark problem for waste collection, such as proposed by Kim et al. in 2006^27^. The ten test difficulties with up to 2092 consumers and 19 garbage disposal sites make up the document. Another group of benchmark issues is a benchmark set created by Crevier et al. for the MDVRPI in 2007^108^. Ten test difficulties approximately up to 288 consumers and seven depots make up this document. These two of the benchmark problems are provided to scientists so they can evaluate any proposed algorithms for solving VRP for waste collecting. The goal of lowering pollution in the waste collection process is only mentioned in a very small number of research. Thus, while providing solutions to the garbage collection problem, the majority of articles concentrate on lowering operational costs and time.

While metaheuristic algorithms have shown great promise in solving solid waste routing problems, they still face some drawback of problem complexity as solid waste routing problems can be highly complex and involve multiple objectives, constraints, and uncertainties^109^. This complexity can make it challenging for metaheuristic algorithms to find optimum solutions, especially when dealing with extensive problems. Metaheuristic algorithms have shown improvements in finding near-optimal solutions for solid waste routing problems. However, they may not always achieve the best solutions or may take longer to converge to a solution than other optimization methods. Additionally, the quality of the solution depends on the problem instance and the algorithm's parameters, which can be challenging to set for a real-world problem. Metaheuristic algorithms may generate solutions that are difficult to interpret. In solid waste routing problems, it is essential to understand the route taken by the waste collection vehicles, as this information can help in making decisions related to resource utilization and environmental sustainability. The lack of interpretability of the solutions generated by metaheuristic algorithms can limit their practical applicability. Solid waste routing problems often have several constraints and uncertainties, such as varying waste generation rates and traffic congestion. Metaheuristic algorithms may not always generate solutions that are robust to these uncertainties, leading to suboptimal solutions or infeasible solutions. This limitation can make it challenging to apply metaheuristic algorithms in real-world situations, where uncertainties are prevalent. Despite of these limitations, metaheuristic algorithms remain a promising approach for solving solid waste routing problems. Researchers are constantly developing new algorithms and improving existing ones to overcome the limitations and enhance their performance.

## Case studies in Asia

A case study review of waste management route problem could involve examining various approaches and strategies used by municipalities or waste management companies to optimize waste collection routes in different cities in Asia. It can provide valuable insights into the current state-of-the-art in route optimization metaheuristic strategies, highlight best practices, and enlighten the future researches and practices in the field.

### Case study 1^110^

**Figure 7:**
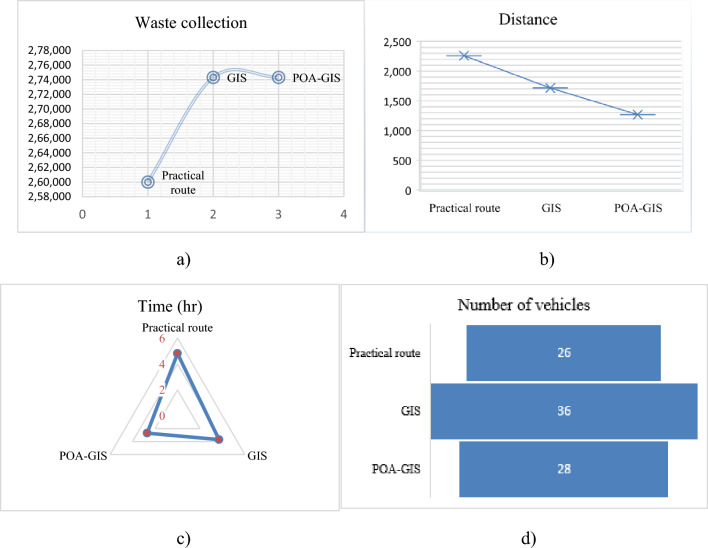
Case study 1: (**a**) total collected waste, (**b**) total travelling distance, (**c**) operation time, (d) number of vehicles

Pelican Optimization Algorithm (POA) combined with Geographic Information System (GIS) software delivers worldwide optimal results and outperforms the traditional techniques. The findings of the research show that planners and engineers can use the developed POA to optimize routing plans, reducing transportation costs and travel distance. It can also be used to reconsider the placement of transfer stations and collection points as constraints variation. The POA is planned with the main aim of simulating the natural hunting behaviour of pelicans. The search agents (pelicans) in this are 90, acting as entities searching for food sources. In order to efficiently handle optimization problems, the mathematical formulation of POA was developed, employing a two-step iteration process to identify the optimal solution. Utilizing the benefits of the algorithm, a real-world example from Ho Chi Minh City, Vietnam was used to find a better solution. Software called a geographic information system (GIS) is typically used to optimize SWC. With this software, real-time road conditions (such as traffic and roadblocks) may be optimized and routes can be scheduled appropriately. Based on the current waste collecting network, the vehicles will carry the waste to the central transfer station before it is disposed of at the landfill. Table 6 summarizes the experimental dataset and outcomes. In terms of total collected waste, travel distance, and operational time, the experimental results are compared with those of real-world routes (Statistics Office of Ho Chi Minh City—District 2 Public Service, 2023) and GIS (Sanjeevi and Shahabudeen 2015). The outcomes from Table 6 and Fig. 7 illustrates the superiority of POA-GIS to other algorithms produce better routes and adapt to real-world situations, maximizing waste collection, minimizing the distance time and number of vehicles, while considering the waste constraint function and confirming all collection sources. Following is the mathematical strategy used in^110^ of CVRP:

**Table 6:** Summary dataset and results.

**Case study: 1**							
Waste sources collection: 247 hotels, 623 restaurants and 230 commercial building Capacity: 10 tons, hook-lift:28							
Objective: Maximize the collection of waste, minimize the total distance, time and number of vehicles used for waste collection							
Optimizing	Practical route			GIS			POA-GIS
Waste collection (kg)	260,000			274,324			**274,324**
Distance (km)	2259			1717			**1269**
Time (hr)	4.8			3.7			**2.7**
Number of Vehicle	26			36			**28**
**Case study: 2**							
Unit vehicle dispatch cost is fixed = 300, transportation cost of unit milage = 10, operation cost handling = 10, cost of unit penalty = 50, permissible error (a) = 2, maximum load (Q) = 30, driving route restrictions (L) = 300, Maximum iteration = 200, ant number = 100							
Objective: Minimizing the cost of vehicle, travelling, handling and time penalty							
Optimizing cost		Traditional strategy				Proposed ACO	
Travel		653,892.6				**651,773.2**	
Handling		**102,778**				**102,778**	
Vehicle		349				**84**	
Dispatch		104,700				**25,200**	
Time penalty		0				**3500**	
Total		861,370.6				**783,251.2**	
**Case study: 3**							
Types of waste disposal points: (1) Landfill (1–5) (2) Mechanical and biological treatment (MBT) (3) Recycling Amount of waste that can be disposed(ton): (1) 7, 6, 5, 7 and 6.8 for landfill (2) 11 and 14 for MBT (3) 10 and 9 for recycling Problem code = C1–C3, No. of trucks = 10, No. of waste disposal sites = 9, No. of types of waste = 3 No. of communities = 22, maximum iteration = 100,000 Objective: lower the fuel consumption							
Technique	Optimizing		C1		C2		C3
Exact solution	Fuel (l)		93.33		55.45		90.27
	Duration (hh:mm:ss)		24:00:00		24:00:00		24:00:00
DE	Fuel (l)		63.20		37.71		66.93
	Duration (hh:mm:ss)		00:08:22		00:07:38		00:08:49
MDE-1	Fuel (l)		57.63		38.20		64.13
	Duration (hh:mm:ss)		00:08:45		00:07:54		00:09:13
MDE-2	Fuel (l)		58.61		39.06		64.24
	Duration (hh:mm:ss)		00:08:59		00:08:10		00:09:21
MDE-3	Fuel (l)		60.64		40.08		65.98
	Duration (hh:mm:ss)		00:08:52		00:08:02		00:09:02

The vehicle's capacity (C) and the amount of waste in the subsequent waste bin determine the model's decision variables, which are represented as follows:

2 $${X}_{ijk}=\left\{\begin{array}{c}1\,if\,vehicle\,k\,can\,travel\,from\,bin\,i\,to\,bin j\\ 0\,Otherwise \end{array}\right.$$

3 $${Y}_{ik}=\left\{\begin{array}{c}1\,if\,vehicle\,k\,can\,travel\,from\,bin\,i\,to\,bin\,j\\ 0\,Otherwise \end{array}\right.$$

The following defines the objective function that seeks to reduce the total collecting distance, Z:

4 $$Z=min\sum_{i=0}^{n}\sum_{j=0}^{n}\sum_{v=0}^{n}{d}_{ij}{X}_{ijk}$$

To make the CVRP model more realistic, the following constraints are considered where N is the number of bins, V denotes the number of vehicles under consideration, C denotes maximum capacity, 0 signifies depot gate, ${d}_{ij}$ is the non-negative distance cost:

5 $$\sum_{i=0}^{n}\sum_{v=1}^{k}{X}_{ijv}=1,\forall j=\text{1,2},...n$$

6 $$\sum_{j=1}^{n}\sum_{v=1}^{k}{X}_{oiv}=1$$

7 $$\sum_{j=1}^{n}{q}_{ojv}=0,\forall v=\text{1,2},...k$$

8 $$\sum_{i=0}^{n}\sum_{v=1}^{k}{X}_{iov}=1$$

9 $$\sum_{j=1}^{n}{{c}_{i}X}_{ijv}\le C\forall j=0,v=\text{1,2},...k$$

10 $$\sum_{i=0}^{n}\sum_{v=1}^{k}{q}_{ijv}- \sum_{i=0}^{n}\sum_{v=1}^{k}{q}_{jiv}={c}_{i},\forall j=\text{1,2},...k$$

11 $$\sum_{j=1}^{n}{X}_{ijv}=\sum_{j=1}^{n}{X}_{jiv}={Y}_{iv},\forall i=\text{0,1},2,...n,v=\text{1,2},...k$$

12 $${dist}_{ij}={dist}_{ji}, \forall i=\text{0,1},2,...n, j=\text{0,1},2,...n$$

13 $${X}_{ijk}\in \{\text{1,0}\}, {Y}_{ik}\in \{\text{1,0}\}$$

where Eq. (5) specifies that bin $i$ is visited by not more than one vehicle $k$, while Eqs. (6) and (7) ensure that a truck begins from the depot and it does not carry any waste. Equation (8) guarantees that, after visiting the last waste bin, a vehicle will reach the depot. Equation (9) shows the collected bin that exceeds the threshold waste level (TWL), in which capacity constraint is a vital problem. Equation (10) signifies that the total amount of waste in a truck cannot exceed its maximum capacity. Equation (11) indicates that a vehicle must fully empty all bins it visits. Hence, the occupied capacity of the vehicle will be equal to the summation of the waste amount of the visited bins. Equation (12) indicates that the distance of two nodes travelled back and forth is the same. Equation (13) signifies the domain of the decision variable. In POA-GIS algorithm incorporate SR1 algorithms as the decoding scheme for the solution representation for the CVRP which has been verified to converge to a global optimum better than other methods.

### Case study 2^111^

A vehicle hybrid scheduling optimization model is introduced, based on the features of recycling and reusing industrial solid waste. This combines VRP with a full load and VRP with a not-full load. The sum of employed vehicle cost, travel cost, handling cost and time penalty cost for waiting or delaying is taken as the objective function in the model. Two constraints of time window and maximal vehicle travel length are introduced in the research. Lastly, an ant colony optimization (ACO) algorithm is presented for solving the problem. Dalian (China) industrial solid waste recycling network is taken as case to validate the efficiency of the proposed model and algorithm. Notations used in this case study is given in Table 7. Table 6 shows the information of study in which by applying the metaheuristic technique, the cost employed of the vehicles reduces by 79,500, the number of employed vehicles reduces by 265, the total cost decreases by 78,119.4 and travel cost reduces by 2119.4. The outcome illustrates that the proposed ACO can search the satisfactory solution quickly and efficiently and proposed model can save the number of employed vehicles and total cost. The mathematical formulation for minimizing the objective function is given below^111^:

**Table 7:** Notations used in case study 2.

Notation	Description
${q}_{i}$	Total amount of waste generated
o	Recycling company
${c}_{o}$	Dispatch cost of each vehicle
${c}_{1}$	Unit running cost of each vehicle
${c}_{2}$	Unit cost of loading and unloading operations
${d}_{ij}$	Distance between the industrial zone i and industrial zone j
m	Number of fleet vehicle
R	Total vehicle trips departing from 0
v	Vehicle speed
Q	Each vehicle’s maximum load
L	Each vehicle’s allowable maximum distance
${t}_{1}$	Time that vehicles reach the industrial area i
${s}_{i}$	Operating time
$E{t}_{i},{LT}_{i}$	Service time window
a	Allowable maximum value of time error
${c}_{3}$	Time window of out of service
${t}_{ij}$	Transport time between two points
L	Longest travel milage of vehicles

Design variable ${t}_{ik}^{r},{X}_{ijk}^{r},{y}_{ik}^{r}$ are:

$${X}_{ijk}^{r}=\left\{\begin{array}{c}1,\,the\,trip\,r\,of\,vehicle\,k\,travel\,from\,i\,to\,j\\ 0,\,otherwise\end{array}\right.$$

Objective function:

14 $$min Z ={c}_{0}\sum_{k=1}^{m}\sum_{j=1}^{n}{X}_{0jk}^{1}+\sum_{i=0}^{n}\sum_{j=0}^{n}\sum_{k=1}^{m}\sum_{r=1}^{R}{c}_{1}{d}_{ij}{X}_{ijk}^{r}+{c}_{2}{d}_{ij}{X}_{ijk}^{r}+{c}_{2}\sum_{i=0}^{n}{q}_{i}$$

$$+\sum_{i=1}^{n}{c}_{3}(max\{E{T}_{i}-{t}_{i}),0\}+max\{({t}_{i}-L{T}_{i}),0\})$$

subject to:

15 $$\sum_{k=1}^{m}\sum_{r=1}^{R}{y}_{ik}^{r}=\left\{\begin{array}{c}\frac{{q}_{i}}{Q}, when\,\frac{{q}_{i}}{Q}\,is\,integer \\ \left[\frac{{q}_{i}}{Q}\right]+1, otherwise\end{array}\right. i=\text{1,2},...n$$

16 $$\sum_{i=0}^{n}{X}_{jik}^{r}=\sum_{i=0}^{n}{X}_{jik}^{r} ; j=\text{1,2},...n, k=\text{1,2},...m, r=\text{1,2},...R$$

17 $$\sum_{i=0}^{m}{X}_{0ik}^{r}\le 1 ; k=\text{1,2}...m, r=\text{1,2},...R$$

18 $$\sum_{i=0}^{m}{X}_{iik}^{r}\le 1 ; j=\text{1,2},...n, k=\text{1,2}...m, r=\text{1,2},...R$$

19 $$\sum_{i\in s}\sum_{j\in s}{X}_{ijk}^{r}\le |s|-1 ;k=\text{1,2},...,m, r=\text{1,2},..,R$$

20 $${y}_{ik}^{r}=\sum_{i=0}^{n}{X}_{jik}^{r}\le 1 ; i=\text{1,2},...n, k=\text{1,2}...m, r=\text{1,2},...R$$

21 $${t}_{ik}^{r}=\sum_{i=0}^{n}{X}_{jik}^{r}\times ({t}_{jk}^{r}+{s}_{j}+{t}_{ji}) ; i=\text{1,2},...n, k=\text{1,2}...m, r=\text{1,2},...R$$

22 $$E{T}_{I}-a\le {t}_{ik}^{r}\le L{T}_{i}+a ;i=\text{1,2},...,n, k=\text{1,2},...,m, r=\text{1,2},...,R$$

23 $$\sum_{i=0}^{n}\sum_{j=0}^{n}\sum_{r=1}^{R}{X}_{ijk}^{r}{d}_{ij}\le L ;k=\text{1,2},...,m$$

where Eq. (14) is set to minimize the whole cost to recycle, the first item related to dispatch cost, the second item related to transportation cost, third item related to handling cost and the fourth item related to waiting cost for time window limit. Equation (15) guarantee the actual service number of vehicle is equal to the number of cars that it needs and according to the insufficient approximation rule the number is rounded. Equation (16) restricts for balance of in and out, ensures that the number of vehicles that arrive and departure from the point in the same car. Equation (17) ensure that each car come from the recycling companies. Equation (18) ensure that each node has one service path and Eq. (19) is a standard branch to eliminate constraints that works for customer collection of vehicle service route. Equation (20) tells the relationship between the two decision variables and Eq. (21) is the arrival time of the trip r of vehicle k when it arrived point $i$. Equation (22) is the time window constraints, when the vehicle arrives at industrial zone in the time window or in the permissible time range of the vehicle can service for industrial zone, it can make the service. Equation (23) ensures that each vehicle road length not exceed the maximum permissible distance L. Considering the constraints of vehicle arrival time and the total time for handling, conditions of non-full load combined with full load operation time limit of recovery that vehicles take can be converted to total mileage limit. The model is suitable for solving vehicle scheduling optimization problem of industrial solid waste recycling after introducing the transportation cost, vehicle dispatch cost, handling and time penalty cost as the objective function. After optimization, from Table 6 and Fig. 8, it can be seen that the proposed ACO is an efficient algorithm to solve the problem.

**Figure 8:**
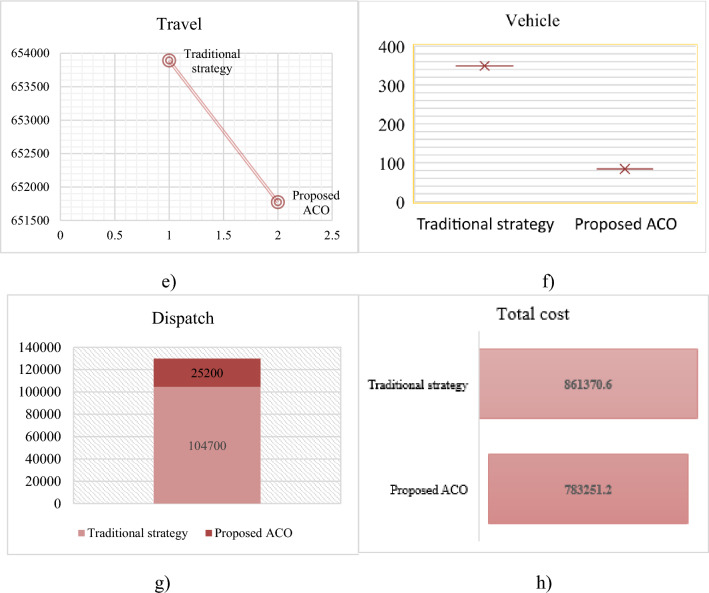
Case study 2: (**e**) travel cost, (**f**) employed vehicle cost, (**g**) dispatch cost, (**h**) total cost.

### Case study 3^112^

**Figure 9:**
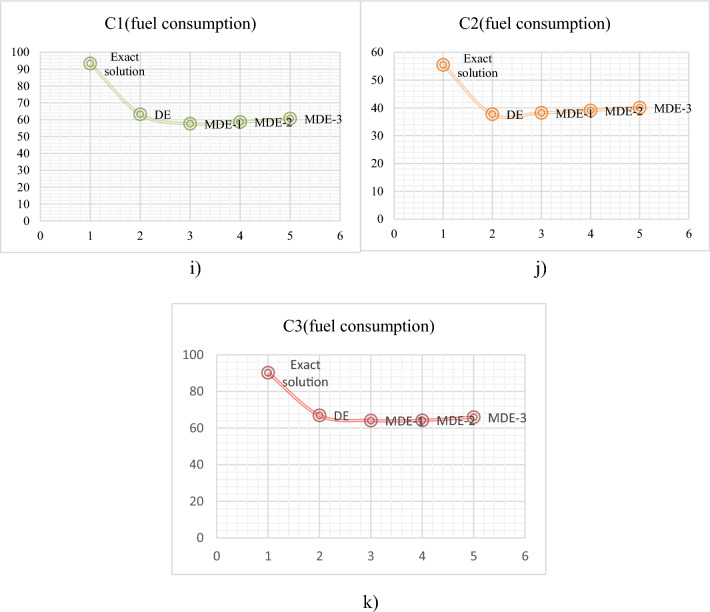
Case study 3: fuel consumption (litres); (**i**) C1, (**j**) C2, (**k**) C3.

In order to transport waste from different sources to different waste disposal points, such as landfills, mechanical and biological treatment (MBT) facilities, and recycling facilities, the research aims to develop a method based on modified differential evolution (MDE). This method will take into account the shortest route to reduce carbon dioxide emissions. A real-life case study of waste transportation in Thailand's Phetchabun Province is used to illustrate the suggested approach. Currently, there is not enough management in place for disposing of waste during the most hectic periods of the year, when more people visit the region. By determining the most efficient path to the disposal location, traditional waste management systems are unable to handle a growing amount of waste. Consequently, the goal of the proposed strategy is to serve as a tool for decision-making to determine the best routes for disposing of waste. The three MDE techniques were used in this study, first was during the recombination phase CR was set at 0.5 and applied the MDE-1 simulated annealing (SA) selection process. In second, the self-adjusting CR value was adjusted from 0.9 to 0.1 in the recombination process and the SA selection process was specified as MDE-2 and in third the recombination process was set up using a primitive selection process that is specified as MDE-3, and the CR value is set to automatically shift from 0.9 to 0.1. Notations used in this case study is given in Table 8. Three models that have been proposed were tested using real information from a case study. This study's vehicle routing problem for waste disposal is a complex one that takes into account a number of variables, including various waste disposal sites, different waste kinds, various truck types, and different speed limitations for different routes. The findings demonstrated that the developed DE provided a solution that satisfied all requirements and constraints and that it could solve the case study problems in less than ten minutes. Table 6 and Fig. 9 indicates that the MDE-1 technique provided the best result (lower fuel consumption), followed by the MDE-2, MDE-3, and DE techniques respectively. Mathematical model formulation is given below for the problem^112^:

**Table 8:** Notations used in case study 3.

Notation	Description
$i$	Set of communities
$j$	Waste disposal points
$t$	Set of trucks
$p$	Set of garbage types
$d$	Truck stop; *d* = 10
${D}_{ij}$	Distance from *i* to*j* (km)
${F}_{t}^{c}$	Fuel consumption rate of the truck *t* (Liters per km)
${F}_{ij}^{r}$	Increased fuel consumption rate when traveling from *i* to *j*
${Q}_{ip}$	Amount of waste type *p* that needs to be disposed of at point *i* (tons)
${V}_{t}$	Maximum loading capacity of the truck at *t* (tons)
${C}_{jp}$	Maximum capacity to dispose of garbage type *p* at waste disposal point *j* (tons)

Design variables:

$${Y}_{i}=\left\{\begin{array}{c}1\,when\,i=1 - 9\,garbage\,disposal\,point\,i,\\ 0\,otherwise \end{array}\right.$$

$${x}_{ijt}=\left\{\begin{array}{c}1\,when\,truck\,t\,travels\,from\,point\,i\,to\,point\,j\,with\,truck t\\ 0\,otherwise\end{array}\right.$$

$${y}_{it}=\left\{\begin{array}{c}1\,when\,truck\,t\,serves\,point i\\ 0\,otherwise \end{array}\right.$$

$${u}_{it}=\text{amount\,of\,garbage\,accumulated\,to\,prevent\,subpath\,formation}(\text{in\,tons})$$

$${v}_{t}=\left\{\begin{array}{c}1\,when\,truck\,t\,serves\,is\,used\\ 0\,otherwise \end{array}\right.$$

$${g}_{jpt}=\text{amount of type p waste that truck t disposes of at point j}$$

Objective function:

24 $$min=\sum_{i\in I}\sum_{j\in J}\sum_{t\in T}{F}_{t}^{c}{F}_{ij}^{r}{D}_{ij}{x}_{ijt }$$

Constraints:

25 $$\sum_{j\in I}{x}_{djt}\le 1 ;\forall t$$

26 $$\sum_{j\varepsilon I}{x}_{ijt}=\sum_{j\varepsilon I}{x}_{jit} ;\forall i,t$$

27 $$\sum_{i\in I}\sum_{p\in P}{Q}_{ip}{y}_{it}\le {V}_{t };\forall t$$

28 $$\sum_{t\in T}{y}_{it}\le 1-{Y}_{i };\forall i$$

29 $${y}_{it}=\sum_{j\in I}{x}_{ijt} ;\forall it\,and\,i\ne d$$

30 $$\sum_{j\in I}\sum_{t\in T}{x}_{ijt}\le \sum_{t\in T}{y}_{it} ;\forall i\,and\,i\ne d$$

31 $$\sum_{p\in P}{g}_{jpt}\le M\sum_{j\varepsilon I}{x}_{ijt} ;\forall jt\,and\,j<d$$

32 $$M{v}_{t}\ge \sum_{i\in I\,an\, j<d }{y}_{it} ;\forall t$$

33 $${v}_{t}\ge \sum_{i\in I\,and\,j<d }{y}_{it} ;\forall t$$

34 $$\sum_{i\in I\,and\,j<d }{y}_{it}{Q}_{ip}=\sum_{i\in I\,and\,j<d }{y}_{jt}{g}_{jpt };\forall pt$$

35 $$\sum_{t\in T }{g}_{jpt}\le {C}_{jp} ;\forall jp$$

36 $${u}_{it}-{u}_{jt}+\sum_{p\in P}{Q}_{ip}\le {V}_{t}(1-{x}_{ijt}) ;\forall ijt\,and\,i\ne j$$

where Eq. (25) guarantees that any truck will leave the truck stop on not more than 1 round, Eq. (26) ensures that any truck traveling to point *i* must leave that point. Equation (27) ensures that each truck will not be transported to the maximum capacity of the load. Equation (28) specifies that the community must be served by at least one truck. Equation (29) specifies that the truck will not be allocated to any *i* community if it is not routed and Eq. (30) ensures that the community is served by passing trucks. The routing of waste transportation from the community to the waste disposal point, or from the waste disposal point to other waste disposal points, will arise when the amount of waste is produced as in Eq. (31). Equation (32) ensures that any truck will travel to service point *i* when that truck is being used. Equation (33) ensures that when any truck is used it must travel to the waste disposal point. The amount of each type of waste that trucks pick up from different communities is equal to the amount of waste brought to the waste disposal point, as specified in Eq. (34). Equation (35) specifies that the amount of waste disposed of at any site *j* must not exceed the maximum waste disposal capacity of that site and Eq. (36) prevent minor path occurrences. The MDE algorithm briefed in the following way: In differential evolution (DE) the first step is creating the initial set, the second step is a mutation procedure and third one is recombination procedure in which the exchange of values from ${U}_{ijG}$ vector trials, has been suggested for the study in order to deliver new outcomes that will be both better and worse than the original results. However, two different CR configurations have been planned, namely a fixed recombination configuration of 0.5, changing the CR in the solution from 0.9 to 0.1 cycles meant that the initial mass exchange was approved, and remains unmodified until the execution cycle is complete (known as adaptive recombination), as in Eq. (37).

37 $${U}_{ijG}=\left\{\begin{array}{c}{V}_{ijG }\,\,if\,rand()\le CR\\ {X}_{ijG}\,\,otherwise \end{array}\right.$$

The three equations are taken into account for picking the target vectors for the subsequent round, the original DE equation calls for selecting the target vector with the best solution out of the old target vectors ${X}_{ijG}$ and the novel target vector. As an outcome of the adjustment of the trial vector ${U}_{ijG}$, where ${X}_{ij(G+1)}$ is the target vector at $i$, the $j$ coordinate in cycle $G+1$ is equal to a vector with a solution value or fitness function that is better between the value of the target vector and the value of the trial vector in cycle $G$. Though, selecting of replies in the subsequent cycle of the DE leads in the possibility of determining low response values locally, or local minima. So, the selection procedure accordingly resolves this problem by adding acceptance of the answer with ${P}_{accept}$ in Eq. (38).

38 $${X}_{ij(G+1)}=\left\{\begin{array}{c}{U}_{ijG }\,\,if\,f({U}_{ijG})>f({X}_{ijG})\\ {U}_{ijG }\,\,if\,rand()>{P}_{accept}\\ {X}_{ijG}\,\,otherwise \end{array}\right.$$

39 $${P}_{accept}=exp-\frac{f({U}_{ijG})-f({X}_{ijG})}{T\cdot K}$$

This study uses the probability in the form of SA from Eq. (39) and the ${P}_{accept}$ value to assess how effective it is to obtain the correct response. The DE and MDE results are displayed in Fig. 9, from which it is clear that the MDE-1 solution is superior to the other case study methods.

## Discussion on case studies

The foremost objective when it comes to waste collection is economical, which aims to reduce costs, time, travel distance, routes, and the number of vehicles involved. Conversely, the environmental component concerns how well the route reduces truck noise, fuel emissions, and the quantity of waste produced. These objectives involve reducing the number of vehicles or resources required, reducing the distance travelled, minimizing the hazards involved in material transportation, reducing the route time, maximizing waste collection, increasing social and environmental profits, and maximizing the compactness of the route. Over two thirds of the researchers concentrated on ways to reduce costs, distance or travel time, and vehicle numbers, while less than one-third of the researchers looked at maximizing route compactness, task balance, environmental emissions, and service quality.

Metaheuristic algorithms can play a more prominent role in optimizing waste management routes and addressing the complex challenges of urban sustainability and environmental stewardship. There are very few case studies related to VRP solution through metaheuristic algorithms for solving WMP in Asia continent and the same situation is persisting in the other continents of the world. In this study we have highlighted the case studies of Vietnam, China and Thailand and the results from the Table 6 and Fig. 9 show the better solution of metaheuristics than the traditional methods. There is a great necessity to explore more metaheuristic algorithms to optimize the WMP alike there are also the other case studies in Asia which do not use metaheuristics to address the problem of WMP.

## Findings and challenges

Metaheuristic algorithms have confirmed their efficiency in solving complex optimization problems, including waste management route optimization. One of the key advantages of metaheuristic algorithms is their capability to be customized and adapted to handle specific constraints and objectives in waste management route optimization. These techniques can include various parameters, such as vehicle capacities, time windows for collection, and environmental considerations, to tailor solutions to specific operational necessities. Geographic Information Systems (GIS) play a vital role in waste management route optimization by providing spatial data on waste generation points, road networks, traffic patterns, and other relevant information. Metaheuristics integrated with GIS information can enhance the accuracy and precision of route optimization solutions by considering spatial constraints and practical conditions.

The fluctuations in waste generation patterns, such as seasonal variations, special events, and population growth, introduce uncertainty into route optimization. Compliance with regulatory requirements, such as waste disposal regulations, vehicle emission standards, and work hour restrictions, poses challenges to route optimization. Choosing the most appropriate metaheuristic algorithm can be challenging for a given waste management route problem and tuning its parameters to achieve optimal performance. Metaheuristic algorithms often require significant computational resources, including computing power and memory, to resolve large-scale optimization problems within reasonable time frames. Conducting case studies with limited computational resources may restrict the size and complexity of the problem instances that can be addressed. By overcoming these complexities, SWM organizations can harness the power of metaheuristic algorithms to optimize route operations, boost service delivery and attain sustainable waste management outcomes**.** It is very essential to conduct case studies by exploring more metaheuristic algorithms to optimize route system of waste management in the countries like India and China which have huge population.

## Conclusion and future scope

Growing world population along with fast economic growth and increased living standards have increased the municipal waste generation making its management be a foremost global issue. In this paper the attempt has been made to survey the recent developments in the vehicle routing problem (VRP) and its variants. The literature is classified into mathematical methods, heuristics approaches and meta-heuristics.

In waste collection problem traditional techniques such as linear Programming (LP), integer Programming and many others guarantee optimal solutions for medium to small sized problems but struggle with large scale instances and do not fit well to large real-life problems because of computational restrictions. They have simple mechanism but can become complex when addressing non-linearities and constraints. Whereas metaheuristics can provide near-optimal solutions for large and complex problems, they are more flexible and can easily adapt to different types of constraints and dynamic environments. Metaheuristics are computationally intensive, tend to offer better performance for large-scale and real-life problems. The literature solidifies its foundational analysis and broadens the scope of understanding regarding metaheuristic algorithmic solutions in waste management. Metaheuristic algorithms offer a powerful toolkit for optimizing waste route problems, enabling waste management organizations to enhance operational efficiency, reduce costs, minimize environmental impact, and improve service delivery to communities. By leveraging the strengths of metaheuristic techniques, stakeholders can make informed decisions and implement sustainable waste management practices that contribute to a cleaner, healthier, and more resilient urban environment. They employ iterative search strategies that explore the solution space iteratively, gradually improving the solution quality over time. This efficacy is particularly beneficial in waste management, where route optimization must be performed regularly to adapt to changing waste generation patterns and operational constraints. Metaheuristic techniques can scale effectively to handle waste route problems of different sizes and complexities. Whether dealing with a small urban area or a sprawling metropolitan region, metaheuristic algorithms can adapt to the problem size and find feasible solutions within a rational timeframe. This scalability makes them appropriate for practical applications where waste management systems may differ in scale and scope.

Metaheuristic algorithms are the powerful tools for solving complex real-life problems, including waste collection. However, they also face limitations such as finding the optimal set of parameters is time consuming and may involve considerable experimentation, maintaining a balance between exploring new solutions and exploiting current ones is challenging and effects the performance and the high computational costs can impose problem for very large datasets.

According to this manuscript, current research is more concerned with enhancing collection procedures efficiency by reducing travel time, distance, and logistical expenses. Some researchers are trying to cut costs by optimising the routes for waste collection vehicles, while others are locating disposal facilities and collection waste bins in the most beneficial locations and also reducing the number of vehicles. The analytical results of the three-case study of Asia for Vietnam, China and Thailand using metaheuristics show the better solutions as compared to traditional methods.

Few of the recommendations for future researches centre on chances to increase the effectiveness of the collection scheme by altering businesses distribution plans, maximising the fleet of vehicles, and incorporating road traffic data into the waste collection systems to prevent causing traffic jams. Meanwhile real information can be tracked and taken into interpretation for scheduling, data driven schemes and novel technologies will aid and contribute in this way. It is also clear from the analysis that there aren't many studies that attempt to combine environmental, social and economic goals. Environmental issues are crucial in contemporary society, particularly in metropolitan regions where trash production is rising along with population. In the future, when attempting to address waste collection using VRP, the economic and environmental goals should be merged. In light of expanding environmental regulations, it would be considered to reduce CO_2_ emissions systematically though efficient route planning. The environmental implications of WMS show that waste material transportation activities are a significant component of the green logistics agenda, particularly in municipal regions with expanding populations. Finally, by utilising VRP and metaheuristic algorithms, this work helps to enhance consideration of the state of the art in garbage collection. This article solely examined solid waste in WMP, however another category of trash, such as polluted and harmful waste, food oil related waste, domestic plastic and medicinal waste, all have unique characteristics. The collection and disposal of these varied wastes should be optimised in coming work. Many real-world waste routing problems are dynamic and change over time due to factors such as traffic, weather, and customer demands. Metaheuristic algorithms can be adapted to solve dynamic routing problems in real-time, where solutions are continuously updated based on new data and conditions. The Internet of Things (IoT) can provide real-time data on waste generation, transportation, and disposal, which can be used to optimize waste routing. Metaheuristic algorithms can be integrated with IoT technologies to develop smart waste routing systems that dynamically adapt to changing conditions and optimize the use of resources. Solid waste routing problems involve several disciplines, including transportation, logistics, and environmental science. Metaheuristic algorithms can benefit from interdisciplinary research, where experts from different fields collaborate to develop new models and algorithms that address the unique challenges of waste routing problems. Hybridization is another aspect which is not much used in waste management route optimization. Hybrid metaheuristic can give more optimal solution as compared to other methods and can be proved as a great advantage to the researchers. In the future work plan, the problem may be solved by doing case study in India by applying metaheuristic technique and incorporating real-life constraints such as vehicle failure, traffic congestion and other environmental related problems and solutions will be produced for optimization purposes. The findings of the present paper may provide direction and a step forward for researchers working on metaheuristic algorithms for waste management route optimization.

## Data Availability

The data that support the findings of this research are available from the corresponding author upon reasonable request.
